# L-Lysine Ameliorates Diabetic Nephropathy in Rats with Streptozotocin-Induced Diabetes Mellitus

**DOI:** 10.1155/2022/4547312

**Published:** 2022-09-12

**Authors:** Faezeh Jozi, Nejat Kheiripour, Maryam Akhavan Taheri, Abolfazl Ardjmand, Gholamreza Ghavipanjeh, Zahra Nasehi, Mohammad Esmaeil Shahaboddin

**Affiliations:** ^1^Department of Clinical Biochemistry, Faculty of Medicine, Kashan University of Medical Sciences, Kashan, Iran; ^2^Institute for Basic Sciences, Research Center for Biochemistry and Nutrition in Metabolic Diseases, Kashan University of Medical Sciences, Kashan, Iran; ^3^Institute for Basic Sciences, Anatomical Sciences Research Center, Kashan University of Medical Sciences, Kashan, Iran; ^4^Institute for Basic Sciences, Physiology Research Center, Kashan University of Medical Sciences, Kashan, Iran

## Abstract

**Introduction:**

Diabetic nephropathy is one of the leading causes of end-stage renal disease worldwide. Uncontrolled hyperglycemia and subsequent production of glycation end-products activate the paths which lead to diabetic nephropathy. The aim of this study was to assess the effects of L-lysine on antioxidant capacity, biochemical factors, kidney function, HSP70 level, and the expression of the *TGFβ*, *VEGF*, and *RAGE* genes in rats with streptozocin-induced diabetes mellitus.

**Methods:**

Thirty-two male Wistar rats were randomly allocated to four eight-rat groups, namely, a healthy group, a diabetic group treated with vehicle (DM + vehicle), a diabetic group treated with L-lysine (DM + Lys), and a healthy group treated with L-lysine (healthy + Lys). Rats in the DM + Lys and the healthy + Lys groups were treated with L-lysine 0.15%. The levels of fasting blood glucose, insulin, HbA_1C_, advanced glycation end-products (AGEs), lipid profile, serum creatinine, blood urea nitrogen, glomerular filtration rate, urine microalbumin, oxidative stress parameters, kidney histology and morphology, and *TGFβ*, *VEGF*, and *RAGE* gene expressions were assessed. *Findings*. An eight-week treatment with L-lysine significantly reduced the levels of fasting blood glucose, AGEs, kidney function parameters, oxidative stress parameters, lipid profile, and the *TGFβ*, *VEGF*, and *RAGE* gene expression and significantly increased the levels of serum insulin and tissue HSP70.

**Conclusion:**

Treatment with L-lysine seems to slow down the progression of diabetic nephropathy.

## 1. Introduction

Diabetes mellitus (DM) is a group of common metabolic disorders characterized by varying levels of disturbance in insulin secretion, dependence on insulin, and increase in blood glucose concentration [1]. Hyperglycemia in DM causes tissue injury and plays a significant role, through different mechanisms, in the pathogenesis of the long-term complications of DM such as cardiovascular disease, nephropathy, neuropathy, retinopathy, and wound healing disorder. One of these mechanisms is nonenzymatic intracellular and extracellular glycation and production of the advanced glycation end-products (AGEs) which are produced through the interaction of reducing sugars with the active amino groups of proteins and other biomolecules [2]. AGEs form covalent cross-links in proteins, lipids, and nucleic acids and thereby make structural and functional changes in biomolecules. The binding of AGEs with the receptor for AGE (RAGE) also forms reactive oxygen species (ROS) and leads to oxidative stress. These processes are the main mechanism for the development of DM complications [3, 4].

AGEs and RAGE are present in kidney cells [5] and play a significant role in the induction of invasion, cell cycle arrest, and proinflammatory changes in kidney cells [6]. AGEs can increase the production of matrix metalloproteinases which in turn cause kidney dysfunction and nephropathy. Besides, AGEs stimulate, through RAGE, the expression of interleukin 6, TNF-*α*, and TGF*β*1 [7]. Stimulation of TGF*β*1 is associated with more production of collagen types I and IV, prevents the expression of proteoglycans, and thereby leads to polymerization and abnormally increases extracellular matrix [8]. Functional and morphological analyses of the kidney in diabetic animals that carried the human AGER transgene showed the acceleration of diabetic nephropathy characterized by albuminuria, glomerular hypertrophy, nephromegaly, and glomerulosclerosis. These changes were not observed in animals without the human AGER transgene [9]. Moreover, the vascular endothelial growth factor (VEGF) plays a key role in inflammation and wound healing through controlling both angiogenesis and vascular permeability [10]. A study showed that in pathological conditions, VEGF binds its receptors in vascular endothelial cells and plays a significant role in angiogenesis [11]. Moreover, VEGF binding to its receptors on endothelial cells stimulates chemotaxis and matrix metalloproteinase production [12].

Clinical evidence shows that reducing AGE formation can significantly reduce the risk of DM complications [13]. There are different strategies to prevent the injuries associated with glycated proteins. Some of these strategies are prevention of protein glycation, use of AGE inhibitors such as aminoguanidine, and induction of protein chaperones to protect the function and structure of proteins [14]. Recently, studies showed that some compounds such as glycerol, trehalose, and L-lysine amino acid can act as chemical chaperones to stabilize proteins and protect them against thermal and chemical denaturation [14, 15]. Some small molecules are the suppressors of protein glycation and influential factors in the alleviation of DM. For example, L-lysine amino acid, as a chemical chaperone, can reduce the nonenzymatic glycation of proteins, protect the native folding of glycated proteins, and increase the levels of heat shock proteins (HSPs) such as HSP70 [16]. HSPs, or stress proteins, protect cells against heat shock, chemicals, induced pathophysiologic stress, and cytotoxic aggregated proteins. HSP70 is a main family of stress proteins which acts as a chaperone molecule and has a significant role in protein folding [17, 18].

L-Lysine has antihyperglycemic effects and can improve diabetic conditions in human and rat through reducing blood sugar and nonenzymatic glycation. Two studies revealed that L-lysine postponed cataract, as a complication of DM, through reducing blood glucose [19, 20]. However, a study showed that a twice daily intake of L-lysine for two months had no significant effects on blood glucose among patients with DM [21]. Besides its actions as a chemical chaperone, L-lysine can reduce inflammation by reducing the levels of interleukin 4 in the kidney and interleukin 10 in the liver [22]. Some studies showed that L-lysine limitation activated NF-*κ*B and ERK1/2 signals and caused inflammation [22, 23]. Moreover, two studies found that the shortage of L-lysine induced apoptosis and caused inflammatory response [24, 25].

Although some studies assessed the effects of L-lysine on blood glucose and some complications of DM, limited evidence exists concerning its effective dose and its molecular mechanisms in diabetic nephropathy. The present study aimed at assessing the effects of L-lysine on antioxidant capacity, biochemical factors, kidney function, HSP70 level, and the expression of the *TGFβ*, *VEGF*, and *RAGE* genes in rats with streptozocin-induced DM.

## 2. Methods

### 2.1. Design

Thirty-two male Wistar rats with an age of eight weeks and a weight of 220 ± 20 grams were housed in controlled temperature with twelve-hour light twelve-hour dark conditions. After three weeks, they were randomly allocated to four eight-rat groups, namely, a healthy group, a diabetic group treated with vehicle (DM + vehicle), a diabetic group treated with L-lysine (DM + Lys), and a healthy group treated with L-lysine (healthy + Lys). Rats in the DM + vehicle and DM + Lys groups received an intraperitoneal injection of streptozocin (Sigma-Aldrich) with a dose of 65 milligrams per kilogram of body weight in sodium buffer with a pH of 4.5 [26]. Rats with a fasting blood glucose (FBG) level of less than 15 mmol/L after three days received another streptozocin injection, and then, rats with an FBG level of more than 15 mmol/L were included in the study. Rats in the healthy and the healthy + Lys groups were treated with vehicle and L-lysine, respectively.

Rats in the healthy + Lys and DM + Lys groups were orally treated with L-lysine 0.15% (Bio Basic Company). Treatment was started one week after DM induction and continued for eight weeks. Urine samples were collected using metabolic cages at the beginning, middle, and end of the eight-week course of treatment. Accordingly, each rat was kept in a metabolic cage for six hours. At the end of the fourth treatment week, capillary blood samples were collected from the eyes using capillary tubes and were used for measuring biochemical parameters. Finally, all rats were sacrificed and heart blood and kidney tissue samples were collected. Blood samples were used to measure HBA_1C_, and serum samples were centrifuged at 3500 g for fifteen minutes and kept at −80°C for further analysis. The Animal Ethics Committee of Kashan University of Medical Sciences, Kashan, Iran, approved this study (code: IR.KAUMS.MEDNT.REC.1397.112), and the experimental protocol of this study was in accordance with the Guideline for the Care and Use of Laboratory Animals approved by this Ethics Committee.

### 2.2. Measurement of Biochemical Parameters

The serum levels of glucose, total cholesterol, triglyceride, high-density lipoprotein cholesterol (HDL-c), creatinine, and blood urea nitrogen (BUN) were measured using enzymatic colorimetric methods (Pars Azmoon Co., Tehran, Iran) [27]. The level of low-density lipoprotein cholesterol (LDL-c) was calculated using the Friedewald formula for samples with a concentration of less than 400 mg/dL of triglyceride [28]. The serum level of insulin was also measured using the enzyme-linked immunosorbent assay (ELISA) kit for rats (Crystal Chem. Company) based on sandwich enzyme immunoassay.

### 2.3. Measurement of Glycation Parameters: HBA_1C_ and AGEs

HbA_1C_ (i.e., glycated hemoglobin) was measured using the high-performance liquid chromatography method, in which glucose-hemoglobin binding was detected through detecting post-glycation structural changes in hemoglobin [29]. As AGE compounds have fluorescence properties, AGE was measured using fluorimetric methods. Accordingly, serum samples were diluted 1 : 50 using 0.1 sodium phosphate buffer (pH: 7.4) and fluorescence intensity was measured at an excitation wavelength of 380 nm and a maximum emission of 440 nm using a spectrophotometer (PerkinElmer/LS55). Results were reported as the percentage of fluorescence emission (F%) [30].

### 2.4. Measurement of Urine Parameters

Urine creatinine was measured using enzymatic colorimetric methods. Creatinine in 24-hour urine was also measured based on urine volume and by collecting 24-hour urine using metabolic cages. The microalbumin content of urine was also measured using the immunoenzymatic ELISA kit (PadtanElm Company, Tehran, Iran).

### 2.5. Analysis of Antioxidant Parameters in Serum and Kidney Tissue

To prepare kidney tissue homogenate, a small 100-mg section of the right kidney tissue was removed and homogenized using liquid nitrogen, lysate buffer, and antiprotease phenyl methyl sulfonyl fluoride.

The FRAP assay is used to measure the antioxidant power based on the reduction of ferric-tripyridyltriazine (Fe^3+^-TPTZ) at low pH to an intense blue color ferrous-tripyridyltriazine complex (Fe^2+^-TPTZ) with a maximum absorption of 593 nm [31]. Superoxidase dismutase (SOD) activity and the contents of malondialdehyde (MDA), nitric oxide (NO), and plasma glutathione (GSH) were measured using the commercially available kits and according to the protocols of their manufacturers. Carbonyl protein level was also measured through a step-by-step protocol, in which 2,4-dinitroophenylhydrazine (DNPH) was used which has the ability to react with carbonyl and form a complex with an absorption of 366 nm [32]. The level of catalase enzyme was manually measured using H_2_O_2_. In this method, ammonium molybdate stops the reaction between catalase and H_2_O_2_, and finally, a yellow complex is formed which has a light absorption of 374 nm [33].

### 2.6. Kidney Histology and Morphometric Examination

Kidney samples were immediately washed using ice-cold normal saline, fixed in 10% formalin, cut into five-micron sections, and stained with hematoxylin and eosin [34]. Then, a histologist assessed the slides for the morphological and inflammatory changes of the glomeruli and tubules using an Eclipse 80i light microscope (with a magnification of ×400). The severity of chronic nephropathy was determined on a 0–5 scale as follows: 0: normal histology; 1: less than 1% inflammatory and morphological changes of glomeruli and tubules; 2: 1%–25% changes; 3: 26%–50% changes; 4: 51%–75% changes; and 5: 76%–100% changes.

### 2.7. Measurement of the Expression of the *TGFβ*, *VEGF*, and *RAGE* Genes

For RNA extraction, 100 mg of kidney tissue was dissolved in 1 ml of TRIzol on ice and was homogenized and centrifuged for ten minutes at 1200 g and a temperature of 4°C. Then, 200 ml chloroform was added to the supernatant and it was centrifuged at a temperature of 4°C for fifteen minutes. Then, 500 mL isopropyl alcohol was added and centrifugation was performed at 1200 g and a temperature of 4°C for ten minutes. Finally, RNA solution was washed with 75% ethanol, centrifuged at 7500 g and a temperature of 4°C for five minutes, and was dissolved in 50 mL of DEPC water. Quantitative analysis of RNA was performed using a NanoDrop spectrophotometer (2000c, Thermo Fisher Scientific, USA), and qualitative analysis of RNA was performed using electrophoresis on agarose gel 1%. After RNA extraction, complement DNA (cDNA) was constructed using the cDNA synthesis kit (Parstous Biotechnology Co., Iran). The annealing temperature of the genes was set using the polymerase chain reaction (PCR) thermal cycler. Primers were designed using the Gene runner and the Oligo 6 software (Table 1). The steps of the thermal cycles were as follows:

1. Initial denaturation of the two strands at a temperature of 95°C for five minutes
2. Denaturation of the two strands at a temperature of 95°C for thirty seconds
3. Annealing of the gene primers at a temperature of 60°C for the *βActin* and the *TGFβ* genes, a temperature of 58°C for the *RAGE* gene, and a temperature of 60°C for the *VEGT* gene
4. Leading temperature of 72°C for thirty seconds for polymerase enzyme; and
5. Final leading temperature of 72°C for five minutes for polymerase enzyme

Finally, RT-PCR was performed and Ct for genes was calculated using the 2 − ∆∆*Ct* equation.

### 2.8. Statistical Analysis

The SPSS software (v. 16.0) was used for data analysis. Groups were compared through the one-way analysis of variance, and findings were presented as mean and standard deviation (mean ± SD).

## 3. Findings

### 3.1. Serum Biochemical Parameters

**Figure 1:**
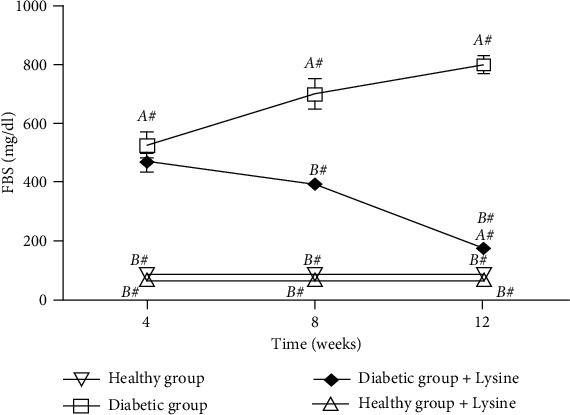
Level of fasting blood glucose in different groups (A#: significant difference between the healthy group and other groups; B#: significant difference between the DM + Vehicle group and other groups).

Following streptozocin injection, rats in the DM + vehicle and DM + Lys group showed an FBG level of more than 15 mmol/L and the typical symptoms of DM such as polyuria and polydipsia. At the end of the study intervention, the level of FBG in the DM + Lys group was significantly less than the DM + vehicle group (*P* < 0.001) (Figure 1). Moreover, STZ-induced DM was associated with decrease in insulin level so that the insulin level in the DM + vehicle group was significantly less than the healthy and the DM + Lys groups (*P* < 0.001) (Table 2).

Streptozocin injection was associated with increase in the levels of HbA_1C_ and AGE. At the end of the intervention, the AGE level in the DM + Lys group was significantly less than the DM + vehicle group (*P* < 0.001), while there was no significant difference between these groups respecting HbA_1C_ level (*P* > 0.05) (Table 2).

Streptozocin-induced DM in the DM + vehicle group was associated with diabetic dyslipidemia, so that the levels of LDL-c, total cholesterol, and triglyceride in the DM + vehicle group were significantly more and the level of HDL-c was significantly less than the healthy group. On the other hand, treatment with L-Lysine was associated with positive changes in the levels of LDL-c and HDL-c, so that the LDL-c level in the DM + Lys group was significantly less and the HDL-c level was significantly more than the DM + vehicle group (*P* < 0.001) (Table 2).

### 3.2. Kidney Function Parameters

Streptozocin injection was associated with alterations in kidney function. In this group, the levels of serum creatinine, serum BUN, and urine microalbumin were significantly more and glomerular filtration rate (GFR) was significantly less than the healthy group (*P* < 0.05). Moreover, the levels of serum creatinine, serum BUN, and urine microalbumin in the DM + Lys group were significantly less than the DM + vehicle group and GFR was significantly more than the DM + vehicle group (*P* < 0.05). Kidney weight at the end of the study in the DM + vehicle and the DM + Lys groups was significantly more than the healthy group (*P* > 0.05), while kidney weight in the DM + Lys group was significantly less than the DM + vehicle group (*P* < 0.05) (Table 3).

Body weight was measured before, four weeks after, and eight weeks after the intervention onset. Group comparisons revealed that the mean of body weight in the DM + vehicle and the DM + Lys groups was significantly less than the healthy group (*P* < 0.001), while there was no significant difference between the rats which received treatment and the rats which did not receive treatment (*P* > 0.05). At the end of the intervention, none of the rats which had received L-lysine showed the side effects of L-lysine therapy such as diarrhea, skin rash, or death.

### 3.3. Antioxidant Parameters in Serum and Kidney Tissue

In the DM + vehicle group, the levels of MDA (as a lipid peroxidation parameter), NO, and carbonyl protein in serum and kidney tissue were significantly more and the levels of TAC, GSH, and SOD and catalase activity were significantly less than the healthy group (*P* < 0.001). In the DM + Lys group, the serum and tissue levels of MDA, NO, and carbonyl protein were significantly less and the levels of TAC, GSH, SOD, and catalase activity were significantly more than the DM + vehicle group (*P* < 0.001). Moreover, the level of HSP70 in kidney tissue in the DM + vehicle group was significantly less than the healthy and the DM + Lys groups (*P* < 0.001) (Table 4).

### 3.4. Expression of the *TGFβ*, *VEGF*, and *RAGE* Genes

**Figure 2:**
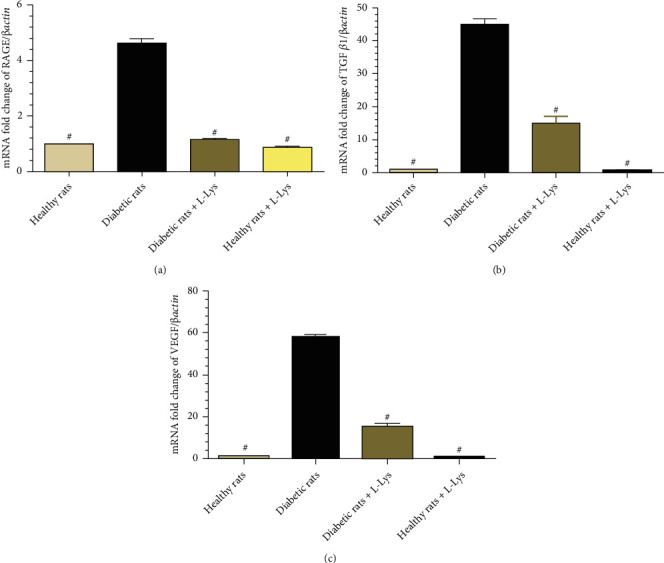
The expression of the RAGE (a), TGF (b), and VEGF (c) genes in kidney tissue (#: significant difference between the DM + Vehicle group and other groups).

The levels of the *TGFβ*, *VEGF*, and *RAGE* gene expression in the DM + vehicle group were significantly more than the healthy group and significantly less than the DM + Lys group (*P* < 0.001; Figure 2).

### 3.5. Kidney Histology and Morphometric Examination

**Figure 3:**
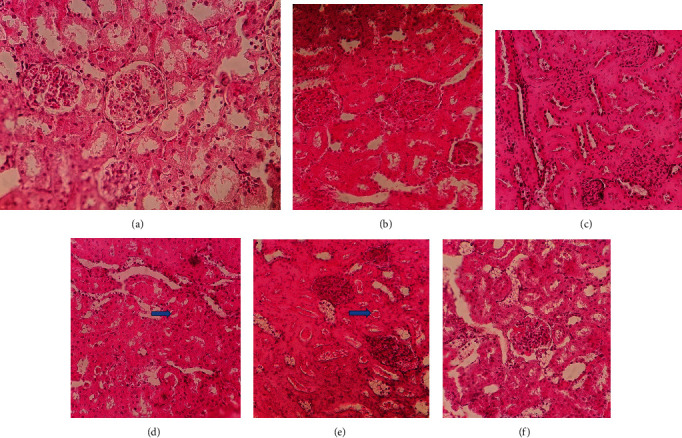
Histological morphology of kidney tissue in the healthy group (a), healthy + Lys group (b), DM + vehicle group (c and d), and DM + Lys group (e and f) (magnification: ×400).

All the rats in both healthy and healthy + Lys groups had normal glomerular morphology (Figures 3(a) and 3(b)). Histological examination revealed increased mesangial matrix, glomerular, and urethral swelling particularly in the proximal tubes, hyaline casts in the urethra, and lymphocyte infiltration (Figures 3(c) and 3(d)). Kidney lesions in the DM + Lys group were also significantly less than the DM + vehicle group (1.08 ± 0.2 vs. 3.25 ± 0.47; *P* < 0.001). Histological examination also showed that treatment with L-lysine significantly reduced kidney tissue injuries in the DM + Lys group (Figures 3(e) and 3(f)).

## 4. Discussion

This study assessed the effects of L-lysine on diabetic nephropathy in rats with streptozocin-induced DM. Findings showed that L-lysine therapy for eight weeks significantly decreased blood glucose and glycation end-products and significantly increased the serum level of insulin. A significant decrease in blood glucose and glycation end-products was observed both four and eight weeks after the onset of treatment with L-lysine. This is consistent with the findings of some previous studies [35, 36]. The catabolism of L-lysine seems to be a significant factor affecting blood glucose level. Amino acids are not stored in the body and are catabolized after exerting their biological (or pharmacological) effects. L-Lysine is catabolized in the liver using *α*-ketoglutarate (*α*-KG) as acceptor and co-substrate. Insertion of more glucose into the liver (insulin dependent entry) to produce more *α*-KG for L-lysine catabolism reduces blood glucose [16]. Two studies reported that D-lysine reduced collagen glycation, serum protein glycation [37], HbA_1C_, and glycated proteins in the lens [38]. Another study found that lysine can significantly reduce collagen glycation [39]. L-Lysine is a chemical chaperone which can stabilize protein structure and protect it against stress without any direct interaction [16]. Moreover, it can bind to free glucose through its amino groups and hence make glucose unavailable to react with other amino groups in proteins [40], which in turn reduces the toxicity of glucose for the body [38].

Our findings also showed a higher insulin level in rats treated with L-lysine. In line with this finding, a study showed that L-lysine supplementation reduced insulin resistance among patients with type 2 DM [41]. Another study reported the positive dose-dependent effects of lysine supplementation on insulin secretion in piglets so that the plasma insulin level increased by 39% when the dose of lysine reached 0.98 of meal weight [42]. Lysine and arginine stimulate insulin secretion through direct membrane depolarization. In this mechanism, positively charged amino acids are transported into *β*-cells, where they depolarize cell membrane and open voltage-dependent calcium channels, which in turn leads to the influx of calcium and secretion of insulin [43]. In the present study, L-lysine might have increased serum insulin level and reduced blood glucose through stimulating insulin secretion from the *β*-cells of the pancreas. Nonetheless, there was no significant difference between the DM + Lys and DM + vehicle groups with respect to the level of HbA_1C_, probably due to the fact that changes in HbA_1C_ level need time. HbA_1C_ is an indicator of blood glucose level in the past 2–3 months, and hence, significant changes in its level usually take three months [44].

The findings of the present study also showed that L-lysine can alleviate hyperlipidemia in diabetic rats. Hyperlipidemia is a common symptom in DM. Continuous renal filtration of lipids in rats with DM leads to chronic kidney injury, albuminuria, and glomerulosclerosis [45]. In agreement with our findings, a study reported that treatment with L-lysine significantly alleviated diabetic dyslipidemia [16]. Another study found that eighteen-week supplementation with essential amino acids plus arginine reduced the levels of plasma triglyceride, total cholesterol, and very LDL-c [46]. In our study, a decrease in the levels of LDL-c and triglyceride was associated with an increase in the level of HDL-c, and hence, L-lysine therapy can be considered as a very effective intervention to prevent cardiovascular disease in patients with DM. On the other hand, we found a significant increase in the FRAP index, as an index of serum antioxidant, in rats treated with L-lysine. Some studies reported that treatment with lysine and antioxidants had positive effects on lipoprotein lipase and LDL-c receptor [16]. Lysine also has a significant role in the biosynthesis of carnitine, which is an essential compound in the transportation of fatty acids into the mitochondrial matrix and their transformation to energy, and hence helps reduce cholesterol, triglyceride, and LDL-c [47, 48].

We also found that L-lysine significantly improved kidney function, increased glomerular filtration rate, and reduced serum creatinine, serum BUN, microalbuminuria, and kidney weight. AGE compounds increase the expression of TGF*β*1 and VEGF growth factors and thereby increase the extracellular matrix, thicken glomerular basal membrane, reduce the elasticity of glomerular capillaries, destroy glomerular capillaries, increase glomerular filtration and permeability and protein excretion in the urine, and eventually lead to diabetic nephropathy [49, 50]. A study found that lysine significantly reduced albuminuria probably through reducing nonenzymatic glycation of collagen in glomerular basal membrane and protecting glomerular integrity [51].

Morphological assessment in the present study also revealed that L-lysine protected kidney tissue and cells against DM. Similarly, previous studies reported that oral administration of L-lysine reduced the serum level of glucose in patients with type 1 DM [52], reduced the glycation of collagen in glomerular basal membrane and albuminuria in rats with DM [51], and reduced the horizontal connections among fibrinogen subunits in patients with type 2 DM [53].

We also found that DM induction was associated with an increase in oxidative stress parameters and decrease in antioxidant parameters. Chronic hyperglycemia leads to the accumulation of AGEs in the body, resulting in the overproduction of ROS and increase in oxidative stress parameters such as MDA and carbonyl protein. ROS damage cell membrane and thereby cause DM complications [38, 49, 50, 54]. Treatment with L-lysine in the present study was associated with an increase in the activity of SOD, catalase, and TAC and a decrease in the levels of MDA, NO, and carbonyl protein in serum and kidney tissue. Similarly, a study showed that dietary lysine improved antioxidant capacity and immunity through TOR and p38 MAPK signaling [55]. Antioxidants reduce the glycation of plasma proteins involved in antioxidant activity such as albumin and hence maintain their functionality. Moreover, they prevent the distribution of transmission metals such as iron and copper which play role in oxidative stress. It seems that lysine not only reduces blood glucose but also inhibits AGE formation, stimulates antioxidant defense, and hence prevents DM complications [56]. Given the significant role of oxidative stress in diabetic nephropathy [57], improving antioxidant capacity using flavonoids, vitamin C, or amino acids such as lysine can prevent or slow down diabetic nephropathy in patients with DM.

Our findings also indicated that streptozocin-induced DM was associated with the high expression of the *TGFβ*, *RAGE*, and *VEGF* genes in rats, while L-lysine administration reduced the expression of these genes. The expression of the *RAGE* gene usually happens in the endothelium, smooth muscles, mesangial matrix, and monocytes. Increased levels of AGEs are associated with increase in the expression of this gene. AGEs and oxidative stress in DM also increase the expression of growth factors such as TGF*β*1 and VEGF [58]. TGF*β*1 and VEGF thicken the extracellular matrix and glomerular basal membrane, reduce the elasticity of glomerular capillaries, and facilitate tissue fibrosis [50]. Compounds such as lysine seem to reduce the expression of *RAGE* and the *TGFβ* genes in kidney tissue and prevent nephropathy by preventing glycation and cross-links in proteins [59, 60]. TGF*β* can start the inflammatory path through ROS [61]. On the other hand, L-lysine prevents hypoxia, formation of free radicals, and activation of inflammatory cytokines through affecting inflammatory cells such as macrophages [62]. A study reported that L-lysine increased microglial M2 polarization and reduced inflammatory response in both in vitro and in vivo conditions, indicating its potential protective effects in the nervous system [25].

Study findings also showed that treatment with L-lysine was associated with an increase in the level of HSP70 and a decrease in the level of AGEs which is in line with the findings of a previous study [63]. This finding denotes that L-lysine can induce HSP70 expression and thereby prevent DM complications in rats. HSP70 is an in vivo marker for evaluating biological response in stressful conditions [64], and its overexpression in the liver, particularly in summer, may positively affect cell survival by providing protection against the changes associated with oxidative stress [65].

## 5. Conclusion

This study suggests the protective effects of L-lysine against diabetic nephropathy in rats with streptozocin-induced DM. Given its positive effects and limited toxicity, L-lysine can be considered as a treatment option to prevent or slow down diabetic nephropathy. Nonetheless, further studies are needed to provide firmer evidence concerning the molecular mechanism of action of L-lysine.

## Figures and Tables

**Table 1:** Primer sequence generated by the gene runner and the Oligo 6 software.

Genes	Forward primer	Reverse primer
*β*Actin	AGGGCTACCATGCCAACTTC	CCACGTAGTAGACGATGGGC
TGF*β*	AGGGCTACCATGCCAACTTC	CCACGTAGTAGACGATGGGC
RAGE	AGAAACCGGTGATGAAGGACA	GGTTGTCGTTTTCGCCACAG
VEGF	CAAACCTCACCAAAGCCAGC	TTCTCCGCTCTGAACAAGGC

**Table 2:** Levels of insulin, HBA_1C_, AGEs, and lipid profile in the whole blood in different groups.

Parameters	Groups			
	Healthy *Mean* ± *S*.*D*.	DM + vehicle *Mean* ± *S*.*D*.	DM + Lys *Mean* ± *S*.*D*.	Healthy + Lys *Mean* ± *S*.*D*.
Insulin (ng/ml)	5.4133 ± 0.39#	0.5925 ± 0.26	3.7175 ± 0.6#	5.2563 ± 0.52#
HbA_1C_ (%)	4.01 ± 0.1#	10.69 ± 0.1	10.27 ± 0.06	3.95 ± 0.08#
AGEs (%)	6.7 ± 4.6#	78.6 ± 7.5	11.2 ± 2#	7.5 ± 0.6#
Triglyceride (mg/dL)	72.46 ± 3.56#	498.92 ± 141.1	99.81 ± 5.25#	59.63 ± 2.46#
Cholesterol (mg/dL)	58 ± 7.13#	188 ± 7.13	56.46 ± 10.5#	58.22 ± 5.97#
HDL-c (mg/dL)	50.55 ± 0.55#	20.56 ± 0.37	58.22 ± 3.99#	54.57 ± 3.23#
LDL-c (mg/dL)	49.34 ± 0.79#	198.67 ± 1.23	56.52 ± 1.34#	46.13 ± 3.15#

#: Significant difference with the DM + vehicle group at a level of <0.001.

**Table 3:** Levels of kidney function parameters in different groups.

Parameters	Time	Groups			
		Healthy *Mean* ± *S*.*D*.	DM + vehicle *Mean* ± *S*.*D*.	DM + Lys *Mean* ± *S*.*D*.	Healthy + Lys *Mean* ± *S*.*D*.
Serum creatinine (mg/dL)	Four weeks	0.75 ± 0.9#	7.40 ± 0.47	5.87 ± 1.04	0.92 ± 0.95#
	Eight weeks	0.766 ± 0.9#	7.71 ± 0.57	1.058 ± 0.7#	0.863 ± 0.27#

Blood urea nitrogen (mg/dL)	Four weeks	24.73 ± 2.32#	115.9 ± 2.06	95.72 ± 1.09	25.95 ± 1.09#
	Eight weeks	24.74 ± 2.32#	165.04 ± 4.53	43.03 ± 1.4#	24.22 ± 3.06#

Glomerular filtration rate (cc/min)	Four weeks	2.64 ± 0.003#	0.045 ± 0.001	1.42 ± 0.24∗	2.66 ± 0.009#
	Eight weeks	3.01 ± 0.003#	0.031 ± 0.002	1.75 ± 0.20#	3.02 ± 0.009#

Urine microalbumin (*μ*g/dL)	Four weeks	60.1 ± 10.52#	663.87 ± 73.9	660.25 ± 70.9	59.90 ± 10.5#
	Eight weeks	62.39 ± 13.58#	1680.98 ± 115.35	740.35 ± 73.98#	60.35 ± 10.5#

Kidney weight (gr)	Four weeks	66.45 ± 11.58#	2045.42 ± 167.92	803.5 ± 10.53#	64.78 ± 11.32#
	Eight weeks	0.71 ± 0.07#	1.37 ± 0.15	1.01 ± 0.09#	0.75 ± 0.07#

#: Significant difference with the DM + vehicle group at a level of <0.001. ∗: Significant difference with the DM + vehicle group at a level of <0.05.

**Table 4:** Serum and tissue levels of oxidative stress biomarkers and HSP70 in different groups.

Parameters	Sample (unit)	Groups			
		Healthy *Mean* ± *S*.*D*.	DM + vehicle *Mean* ± *S*.*D*.	DM + Lys *Mean* ± *S*.*D*.	Healthy + Lys *Mean* ± *S*.*D*.
Malondialdehyde (MDA)	Serum (*μ*mol/L)	3.08 ± 0.57#	7.21 ± 0.23	3.14 ± 0.42#	2.79 ± 0.55#
	Tissue (*μ*mol/mg protein)	2.8383 ± 0.38#	7.015 ± 0.127	2.0064 ± 0.59#	2.785 ± 0.45#

Nitric oxide (NO)	Serum (*μ*mol/L)	0.055 ± 0.011#	0.157 ± 0.009	0.079 ± 0.021#	0.06 ± 0.011#
	Tissue (*μ*mol/mg protein)	0.078 ± 0.008#	0.187 ± 0.007	0.077 ± 0.028#	0.075 ± 0.018#

Superoxidase dismutases (SOD)	Serum (U/L)	90.73 ± 1.85#	28.53 ± 2.53	53.11 ± 2.67#	89.18 ± 2.31#
	Tissue (U/mg protein)	65.01 ± 2.19#	22.72 ± 1.801	50.05 ± 8.09#	64.13 ± 3.42#

Catalase	Serum (KU/L)	22.28 ± 0.73#	9.42 ± 1.56	20.45 ± 1.23#	24.003 ± 2.31#
	Tissue (KU/mg protein)	30.34 ± 0.7#	16.58 ± 1.84	26.86 ± 4.18#	29.9 ± 1.307#

Total antioxidant capacity (TAC)	Serum (*μ*mol/L)	0.23 ± 0.017#	0.15 ± 0.0018	0.22 ± 0.021#	0.22 ± 0.022#
	Tissue (*μ*mol/mg protein)	0.152 ± 0.0109#	0.092 ± 0.009	0.1508 ± 0.012#	0.16 ± 0.014#

Glutathione (GSH)	Serum (mmol/L)	0.086 ± 0.015#	0.002 ± 0.001	0.053 ± 0.011#	0.08 ± 0.013#
	Tissue (mmol/mg protein)	0.108 ± 0.022#	0.017 ± 0.009	0.074 ± 0.015∗∗	0.08 ± 0.007#

Protein carbonyl	Serum (*μ*mol/L)	11425.32 ± 2.64#	15456.85 ± 2.95	13256.11 ± 2.54#	10432.52 ± 1.8#
	Tissue (*μ*mol/mg protein)	9212.31 ± 2.64#	13254.56 ± 2.9	10542.32 ± 2.54	9134.52 ± 1.8#

HSP70	Tissue (ng/ml)	25.60 ± 1.62∗∗	15.92 ± 1.49	30.008 ± 6.18#	26.02 ± 1.20∗∗

#: Significant difference with the DM + vehicle group at a level of <0.001. ∗∗: Significant difference with the DM + vehicle group at a level of <0.01.

## Data Availability

Data are available on request.
